# Coenzyme B12 synthesis as a baseline to study metabolite contribution of animal microbiota

**DOI:** 10.1111/1751-7915.12722

**Published:** 2017-06-14

**Authors:** Antoine Danchin, Sherazade Braham

**Affiliations:** ^1^ Institute of Cardiometabolism and NutritionHôpital de la Pitié‐Salpêtrière47 Boulevard de l'Hôpital75013ParisFrance; ^2^ AMAbiotics SAS47 rue de Montmorency75003ParisFrance

## Abstract

Microbial communities thrive in a number of environments. Exploration of their microbiomes – their global genome – may reveal metabolic features that contribute to the development and welfare of their hosts, or chemical cleansing of environments. Yet we often lack final demonstration of their causal role in features of interest. The reason is that we do not have proper baselines that we could use to monitor how microbiota cope with key metabolites in the hosting environment. Here, focusing on animal gut microbiota, we describe the fate of cobalamins – metabolites of the B12 coenzyme family – that are essential for animals but synthesized only by prokaryotes. Microbiota produce the vitamin used in a variety of animals (and in algae). Coprophagy plays a role in its management. For coprophobic man, preliminary observations suggest that the gut microbial production of vitamin B12 plays only a limited role. By contrast, the vitamin is key for structuring microbiota. This implies that it is freely available in the environment. This can only result from lysis of the microbes that make it. A consequence for biotechnology applications is that, if valuable for their host, B12‐producing microbes should be sensitive to bacteriophages and colicins, or make spores.

## Introduction

**Figure 1:**
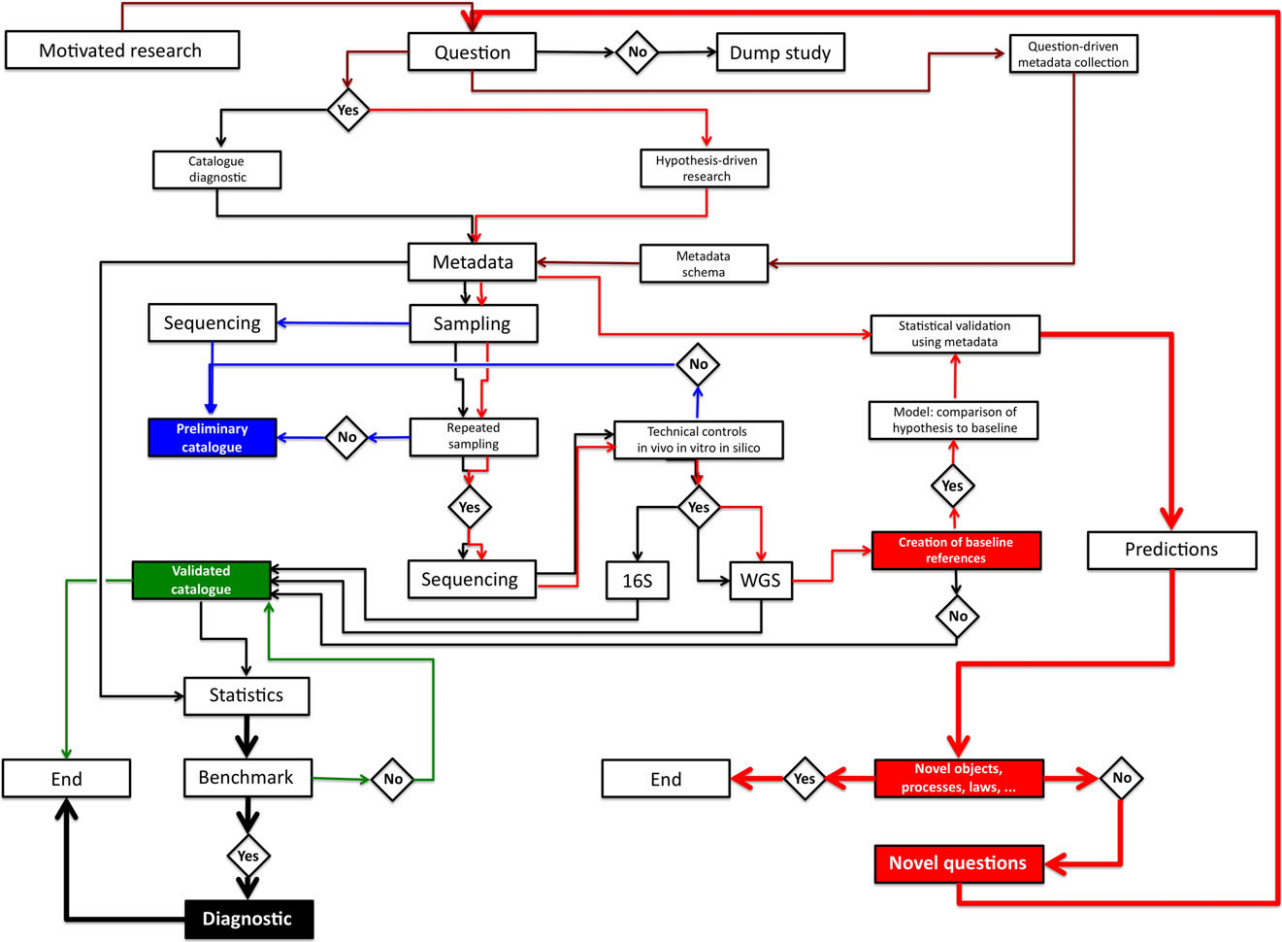
Flow chart of microbiota metagenome studies. Data‐driven research is often purely descriptive. It must start with a specific and clearly stated question, often driven by motivated research (Danchin, 2010). With relevant controls, it may lead to a preliminary catalogue that should be taken with a grain of salt (blue), a validated catalogue (green) or a diagnostic tool (black). Hypothesis‐driven research will require repeated experiments, control experiments, baseline references (the subject of the present article) and model‐building. This will lead investigators to make predictions. If they predict a novel object or process the question can be considered as answered. If not the prediction generates novel questions and the exploration is recursively started again, in a constructive development, typical of what science should be. An essential part of the process is the creation and management of metadata that are linked to the sampling procedure. Connection with the metadata allows investigators to place their questions and answers in relevant context. Sequencing is key to microbiome studies. Here, we considered that restricting sequencing to 16S targets will restrict the study to catalogues and diagnostic. This approach can seldom be used in hypothesis‐driven research, where whole‐genome sequencing (WGS) must be the rule.

The fashionable study of animal metagenomes keeps generating a huge variety of experiments that describe correlations between specific microbiota of animal origin (most often human) and health or longevity. In these studies, many metabolites are suggested as possibly involved in promoting well‐being. Yet, it is exceptional that these studies can separate between recognized effects caused by the microbiota, or consequences of a common cause that acts in parallel on host health and microbe‐dependent metabolite production. To progress, we must compare the features of interest with relevant baselines, where a strictly microbial compound would be known to have an essential contribution to the host well‐being, while exploring how it fares between the microbiota and the host. To proceed towards establishing causality, these baselines must be connected to extensive metadata that describe the nature of the samples and their hosts, using an organized set of descriptors that will be later linked to the outcome of the study (Fig. 1). This may establish an explicit role (possibly direct but often indirect) of the microbiota. Here, we propose an example of such a baseline. The ubiquitous beneficial association between animals and bacteria has long been recognized. Essential cofactors of animals such as coenzyme B12 [vitamin B12 family, cobalamins (Degnan *et al*., 2015)] can only be synthesized by a small catalogue of prokaryotes, but neither by plants nor animals. The model animal *Caenorhabditis elegans* grown under B12‐deficient conditions for five generations (15 days) developed severe B12 deficiency associated with various phenotypes that include infertility, growth retardation and reduced lifespan (Bito and Watanabe, 2016). In *Homo sapiens,* vitamin B12 is required for essential enzymes. Cytosolic methionine synthase recovers homocysteine (toxic when present above a threshold) to make methionine, and mitochondrial methylmalonyl‐CoA mutase scavenges methylmalonate derived from catabolism of branched‐chain amino acids into succinate (Kohl and Carey, 2016). In mitochondria, cobalamin adenosyltransferase, together with an unidentified cobalamin reductase, catalyses the reduction in cobalamin into the deoxyadenosyl form (AdoCbl). Vitamin B12 deficiency is linked to anaemia, developmental disorders, metabolic abnormalities and neuropathy (Shipton and Thachil, 2015).

How does the compound enter the host metabolism, is this a direct product of its own microbiota, an indirect supply via its food or both? A host and its microbiota assemble a metabolism made of diverse pathway branches, constituting a complex meta‐metabolism (Ibrahim and Anishetty, 2012). Metabolite precursors and by‐products are synthesized via collaboration between a variety of organisms that shape the microbiota. A key symbiotic relationship is thus established, contributing to the healthspan of the animal host, via the commensal microbes with which it has co‐evolved (Kohl and Carey, 2016). These microorganisms, located mainly in the gut, but by no means only there, develop on a steady supply of nutrients (provided via the food supply to the host), and in turn provide different functions such as protection against pathogens, activation of the immune system, metabolization of sterols or synthesis of vitamins. It is expected that the contribution of microbiota will considerably differ in different organisms, depending on the structure of their gut. Here, we propose tracks to investigate the contribution of microbiota to coenzyme B12 availability as a baseline for exploring the possible role of other important microbial metabolites.

## Where do hosts get their coenzyme B12?

**Figure 2:**
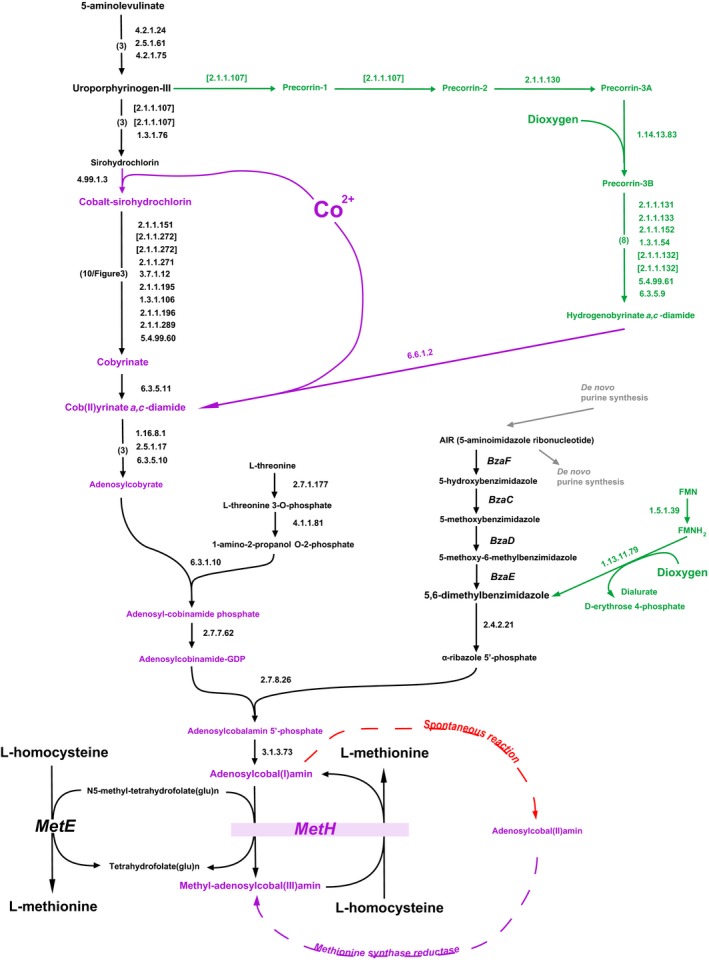
An overview of coenzyme B12 biosynthesis. The anaerobic route, present in gut microbiota, is displayed in black and the aerobic route in green, with the two entry points for dioxygen as indicated. Metabolites comprising a cobalt atom are in purple. Synthesis of methionine is also displayed. The B12‐dependent methionine synthase is reactivated when cobalt is accidentally converted into the cobalt(II), instead of its d^6^ low spin stable form (Danchin, 1973), after a few thousand catalytic cycles (Drennan *et al*., 1994).

Compounds of the coenzyme B12 family (cobalamins) are exclusively synthesized by prokaryotes. Terrestrial plants do not make nor use cobalamins and they have no cobalamin‐dependent enzymes. In these organisms, methionine synthesis uses methionine synthases as some bacteria do, with methyltetrahydrofolate as the sole immediate methyl donor to homocysteine (Ravanel *et al*., 2004). This is a noteworthy case of a balance between different trade‐offs. In the absence of coenzyme B12, transfer of a methyl group from tetrahydrofolate to the sulfur of homocysteine is fairly inefficient, requiring complicated conformational rearrangements (Matthews *et al*., 2003; Pejchal and Ludwig, 2005). As a result, the total amount of MetE, the corresponding methionine synthase, becomes a significant burden for the translation machinery. By contrast, B12‐dependent methionine synthase MetH is considerably more efficient, limiting the amount of the protein requirement. This enzyme is also reactivated efficiently by a specific reductase that restores activity after accidental electron losses during catalysis (Wolthers and Scrutton, 2009). The consequence is that in many bacteria, when *metH* is present, *metE* expression is repressed by a B12 riboswitch (Kazanov *et al*., 2007). However, as seen in Fig. 2, B12 synthesis, salvage or transport requires many genes, which creates a heavy genetic burden that plants solved using the first solution. Following a different track, cobalamin auxotrophy has arisen numerous times throughout evolution. Even in the prokaryotic world, there is only a fairly limited number of organisms synthesizing the molecule, often having lost the vitamin B12‐independent form of methionine synthase, while requiring association with commensal communities producing B12. As cases in point, some important algae recruited the B12‐dependent enzyme, acquiring B12 through symbiotic association with bacteria (Croft *et al*., 2005; Kazamia *et al*., 2012) and then lost their B12‐independent enzyme (Helliwell *et al*., 2011). Interestingly, even though plants do not make or use B12, nodule‐forming *Sinorhizobium meliloti* requires a cobalamin‐dependent ribonucleotide reductase for symbiosis with its plant host (Taga and Walker, 2010).

### Insects

Insects form the largest category of plant‐eating animals. In early work, vitamin B12 failed to be detected in many diverse insect species (Halarnkar and Blomquist, 1989). However, a positive bacterial contribution is obvious in cockroaches, where the commensal *Shimwellia blattae* has a full counterpart of a B12 synthesis pathway (Brzuszkiewicz *et al*., 2012). It is further known that *Aedes aegypti* codes for a B12‐dependent methionine synthase (Jaffe and Chrin, 1979), but it may have obtained the coenzyme via its bloodmeal and eventually transmitted it to its progeny, including males that do not feed on blood. It is, however, noteworthy that germ‐free mosquitoes fail to undergo full development and die, demonstrating an essential role of microbiota in these insects (Coon *et al*., 2014). Skin microbiota, producing L‐lactate in particular, attract malaria mosquitoes [references in Mweresa *et al*. (2016)], possibly driving mosquitoes to B12‐making microbial sources. To be sure, a likely major role of vitamin B12 is prominent in notable insects. Termites display high levels of vitamin B12, presumably synthesized by endosymbiotic microorganisms, or possibly gut microbiota [known to be rich in Archaea (Brune and Dietrich, 2015)]. Despite having the smallest gene set known in bacteria, the cicada symbiont *Hodgkinia* devotes at least 7% of its proteome to cobalamin (vitamin B12) biosynthesis, a significant metabolic burden (McCutcheon *et al*., 2009). In general, we miss studies focusing on the importance of B12‐dependent enzymes in insects (Zhang *et al*., 2009) and much has still to be investigated in relation with their often rich microbiota. Exploration of the domain began developing just before year 2010 and it is now growing steadily, with *Drosophila melanogaster* as a convenient model (Leulier *et al*., 2017).

### Fishes

As a relevant metadata linked to microbiome studies, the lack of B12 in plants is an incentive to segment the members the vertebrate class according to their feeding habits: herbivorous, omnivorous and carnivorous. Because carbon fixation is performed by plants, herbivorous vertebrates make the vast majority of their class. There is such a variety of those that we can only get coarse ideas about the way their metabolism is organized in collaboration with their microbiota. Among vertebrates, fishes comprise a majority of herbivorous species. However the plants they thrive on are essentially algae, and because many algae species get their essential B12 complement from microbiota, it is likely that the contribution of fish commensal microbes is highly variable, if not negligible, as indeed observed when fish is supplied with industrial food (Bogard *et al*., 2017). The outcome of this situation is that fishes living in wild environments are expected to be a rich source of B12 (Braekkan, 1958) for carnivorous and omnivorous species (this might be an amusing explanation for the behaviour of fish‐loving, water‐hating cats, and for that of grizzly bears). Some fishes live in symbiosis with phytoplankton and cyanobacteria that produce toxins protecting them against predators (Tosteson, 1995). By themselves, cyanobacteria might be good sources of B12, although the cobalamin variants they produce often are inactive or act in mammals as antimetabolites, rather than vitamins (Watanabe *et al*., 2013).

### Birds

In general reviews of their association with microbial studies, vertebrates of various feeding habits, cattle, cetaceans, reptiles or birds, are known to require B12 for a variety of enzymes (Hanning and Diaz‐Sanchez, 2015). The question that arises again is then whether the microbiota associated with a particular host produce enough of the molecule for steady‐state use, or whether the host needs to complement this supply via eating meat from self‐sufficient animals (via their microbiota) for the production of the molecule. Birds need B12. It has long been observed that pigeons or chickens fed only seeds suffered pernicious anaemia, caused by B12 deficiency. There was, however, a considerable variation between individuals, as expected if there was at least some contribution of individual microbiota (Castle, 1985). Antibiotic supplementation, a long used while disputable practice, increases the meat yield of poultry, while affecting microbiota, especially in increasing the proportion of lactobacilli (Crisol‐Martinez *et al*., 2017). Antibiotic‐dependent promotion of *Lactobacillus reuteri*, previously isolated from many vertebrates [mouse, rat, man, pig and chicken (Frese *et al*., 2011)], and which has many B12 synthesizing strains (Spinler *et al*., 2014), may be a case in point.

### Mammals

Endowed with large complex microbiota, polygastric mammals, which have a gut structurally apt to allow microbial development, are stably rich in vitamin B12 (Ortigues‐Marty *et al*., 2005). They make a very important category of animals that provide the bulk of the B12 needed by terrestrial carnivorous and omnivorous mammals. This ability has been shown to rest on cobalt availability in their environment, indirectly demonstrating that their B12 content indeed depends on their microbiota (Al‐Habsi *et al*., 2007). As a consequence, milk, which is rich in cobalamins, will display variable amounts of the molecule. The horse, while monogastric, does not appear to suffer B12 deficiency (Roberts, 1983). The gastric mucosa of this animal is rich in bacteria (Perkins *et al*., 2012), some of which likely to produce cobalamins. It would be of interest to explore more in‐depth horse metagenomes to identify possible sources of cobalamins. Omnivorous pigs, fed on plant feed, exhibit B12 deficiency, suggesting that their own microbiota does not provide a sufficient amount of the coenzyme. Recent studies show that supplementation might have a positive effect during reproduction (Simard *et al*., 2007). Rodents are often vegetarians, but they indulge in occasional animal food, which may compensate for cobalamin deficiencies. The healthy status of their B12 supply has not been investigated, except for commensals such as rats (Khaire *et al*., 2017) or mice (Ghosh *et al*., 2017), where, as discussed below, B12 synthesis is most likely related to their microbiota. Non‐human primates have generally a large gut (another noteworthy piece of metadata), fitting with their mostly plant‐eating habits (Milton, 1987). Studies of their microbiomes have begun fairly recently, in particular under field conditions (Hale *et al*., 2016). While human feeding habits are mostly of the omnivorous type, with usually sufficient cobalamin availability, human vegetarians display a highly variable B12 store. This indicates that the contribution of their microbiota is limited, if it even exists on a general basis [see (Rizzo *et al*., 2016), and below, for a preliminary study of human populations (Braham, 2015)]. The origin and fate of cobalamin availability are therefore expected to provide a valid baseline for the possible microbial input of other important micronutrients.

A common habit of many of these animals – man excepted – is that they find ways to create a supply of many essential nutrients and rich microbiota, coprophagy (Soave and Brand, 1991), as we now see.

## Behaviour and time in relation to B12 availability

Our deeply anthropocentric views (we might perhaps say ‘victorian’ attitude) make that we often fail to recognize the importance of behaviours that have considerable consequences in terms of propagation and role of microbiota. Coprophagy is a case in point. Egyptian vultures, *Neophron percnopterus*, obtain carotenoids (and presumably many other important metabolites as well) from eating the excrement of ungulates (Negro *et al*., 2002). Many carnivorous animals are also attracted by the entrails of their preys.

Another misleading while somewhat ignored contribution to the role and recruitment of microbiota is that they are very sensitive to the age of their host. Let us see how this may fit with B12 availability.

### Coprophagy

Faeces are obvious sources of large microbiota, yet neglected in most studies. Coprophagy is expected to be an important behaviour if microbiota supply complex metabolites such as cobalamins (Rosenberg and Zilber‐Rosenberg, 2016). This is distinctly relevant when a substantial fraction of the microbial organisms thus ingested is degraded – we will reiterate this point. Microbe degradation releases their cytoplasmic content in the stomach, with its generally acid composition coupled to synthesis of cobalamin‐binding proteins [intrinsic factor (Alpers and Russell‐Jones, 2013)]. Rodents, lagomorphs and to a lesser degree piglets, foals (this may account for B12 availability in the horse), dogs and non‐human primates engage in coprophagy. This behaviour appears to be necessary to supply essential nutrients or microbes. To be sure, microbial synthesis of valuable metabolites occurs in the lower gastrointestinal tract in these animals where little absorption is realized. For example, rabbit pups ingest faecal pellets of their mothers (Combes *et al*., 2014). Eating their own faeces is a behaviour with strong selective advantage for picking up these nutrients, B12 in particular, while seeding their gut with beneficial microbes (Soave and Brand, 1991). In laboratory experiments using rodents, coprophagy should be monitored carefully, as it might considerably alter the meta‐metabolism of microbiota (Ohta *et al*., 1996). In primates, coprophagy is often treated as abnormal (in zoo) (Jacobson *et al*., 2016), while in fact it is a normal behaviour in the wild (Sakamaki, 2010). The main negative outcome of coprophagy is that it is a very efficient way to propagate parasites (Walsh *et al*., 2013). This may account for the generally coprophobic behaviour of man, an animal which had the opportunity to eat a substantial amount of animal food, thus depending less on its own microbiota, as we shall see.

### The role of age

As their hosts do, microbiota evolve with age, and B12 deficiency is often a marker of ageing in animals. We have discussed the general structure of fish microbiota, but it is certainly sensitive to fish development, varying with time (Li *et al*., 2017). Microbiota of ruminants also change significantly with host age, as demonstrated in *Bos grunniens*, the yak (Nie *et al*., 2017). Investigation of age‐dependent bovine rumen isolated from five age groups, from 1‐day‐old calves to 2‐year‐old cows, revealed changes occurring after birth, reflected by a decline in aerobic and facultative anaerobic taxa and an increase in anaerobic ones (Jami *et al*., 2013). Microbiota of mice is modulated both by food and by age (Tachon *et al*., 2012), and metabolomics studies unveiled an ageing microbial signature in mice (Calvani *et al*., 2013). In another study, it was found that ageing mice overexpressed specific bacterial clades, such as the *Alistipes* bacterial genus. In parallel, cobalamin and biotin biosynthesis was decreased (Langille *et al*., 2014). Intestinal uptake of B12 is also modified with age, with lower transport in aged rat enterocytes, for example (Toyoshima *et al*., 1983). This has important consequences for carnivorous animals because the input of cobalamin will depend on prey age, a parameter that is seldom taken into account in the metadata of microbiome metagenome studies. For example, hen's eggs, considered good supplies of B12, have a decreased amount of the coenzyme as the hens become older (Robel, 1983). Whereas this is caused by the host ageing or by that of its associated microbiota needs to be investigated. The duck microbiome also changes with age (Best *et al*., 2016). An important consequence is that when doing microbiome studies it is essential to compare individuals with matching ages and probably to monitor the age of the animal food they eat, as aged food may impact the ageing process itself (Lee *et al*., 2017).

Man is no exception in this respect. Investigation of the composition of microbiomes from newborns to centenarians showed that subjects’ microbiota were divided into two broad age‐related groups, the adult‐enriched and infant/elderly enriched clusters (Odamaki *et al*., 2016). In man, conventional microbiological investigations of the faecal microbiota showed both bacteria‐specific as well as a general pattern of ageing of the colonic microbiota, with the last decades (older than 60 years) demonstrating the most profound changes. Whether these changes reflect direct changes of the gut microbiota, the mucosal innate immunity or indirect consequences of age‐related altered nutrition is still to be investigated (Enck *et al*., 2009).

A major consequence of this variation of microbiome composition as a function of age is that metadata associated with any statistical approach meant to explore the details of microbiome structure should be stratified as a function of the hosts’ age. This is particularly important for clustering methods that will be misled and might create spurious classes by likely interference with age.

## Signatures of coenzyme B12 biosynthesis

In man as in other animals, the supply of vitamin B12 depends on the diet, but may also, to a limited extent, depend on the gut bacteria. However, this latter contribution, in non‐coprophagic animals, is limited because the pathway of B12 absorption asks for acid‐dependent, intrinsic factor‐dependent transport, linked to transit through the stomach (Alpers, 2016). Substantiating this point, patients who have been submitted to bariatric surgery are generally B12 deficient (Dogan *et al*., 2017). Following absorption, cobalamin is stored in significant quantities in the liver to be used daily by the body. This standard store of cobalamins is sufficient to fill human needs for a period of up to 3–5 years. As a consequence, if dietary intake is inadequate, clinical signs of deficiency are not manifest until years after the exhaustion of resources. This has considerable impact when monitoring the health of persons who changed their diet. In the case of *Homo sapiens* living in industrial countries, the synthesis of the coenzyme by the human microbiota [less than half of the marker genomes of the human gut are able to produce cobalamin, restricted to the bacteria themselves anyway (Magnusdottir *et al*., 2015)] is insufficient to meet the typical needs of the organism. This implies that B12 (obtained from the microbiota of other organisms) is present in significant quantities in animal protein food. It is therefore worth exploring whether the composition and management of diverse microbiota provide hints about how, where and when B12 is made and used. This prompted a study that compared microbiota from humans living in traditional societies with microbiota produced in industrial countries (Braham, 2015).

Looking for gene signatures is a direct way to explore the functional capacity coded by microbiomes. As a recent case in point, in rumen, known to rest on consortia of microbes, the signature of the *luxS* gene that codes for synthesis of autoinducer‐2 (a general quorum sensing trigger involved in assembling microbial communities) was used as a probe to monitor the rumen structure (Ghali *et al*., 2016). In the same way, exploring microbiomes for their ability to direct an efficient coenzyme B12 synthesis asks for identification of specific signatures of key genes in the biosynthetic pathways. Cobalamin is comprised of three parts: a tetrapyrrole core (the corrin nucleus), with four nitrogen atoms liganded to a cobalt ion, and two axial ligands, the alpha ligand bound to a pseudo‐nucleotide group, 5,6‐dimethylbenzimidazole (DMB) and the beta ligand, involved in catalysis, made of a variable radical in position beta which is used to name the cobalamin variant [‐CN for cyanocobalamin, ‐OH for hydroxocobalamin, ‐CH_3_ for methylcobalamin (methylation donor in the formation of methionine from homocysteine) and 5′‐deoxyadenosylcobalamin (cofactor of methylmalonyl‐CoA mutase)]. Among the many types of corrinoid B12 derivatives, the chemical form commonly synthesized by microorganisms is adenosylcobalamin.

**Figure 3:**
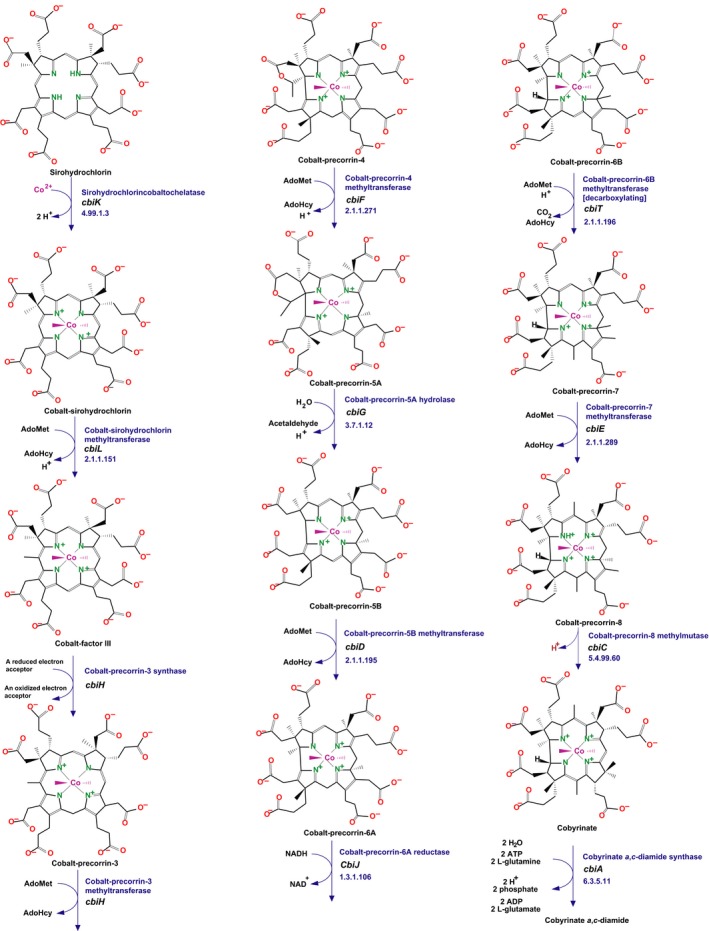
Central section of anaerobic synthesis of coenzyme B12. This part of B12 metabolism involves the cobalt atom at the centre of the corrin ring (octahedral coordination sphere). Signatures of the corresponding proteins are displayed in Table 1.

Many genes are involved, making the signature exploration endeavour plausible. Two major routes drive the biosynthesis of the corrin ring (Caspi *et al*., 2016): an anaerobic route, most of which characterized in *Salmonella typhimurium* (Moore and Warren, 2012; Hazra *et al*., 2015) and an aerobic route identified in *Pseudomonas denitrificans* (Ainala *et al*., 2013). These routes differ in their requirement for dioxygen and the timing of cobalt insertion (Fig. 2). A monooxygenase of the aerobic pathway requires dioxygen to facilitate the ring contraction process prior to cobalt insertion, whereas the anaerobic route, the only one important for gut microbiota, inserts cobalt early, before ring contraction (Fig. 3). The routes diverge and then converge at the synthesis of cob(II)yrinate *a*,*c*‐diamide, followed by a very similar pathway. Finally, synthesis of an axial ligand, usually 5,6‐dimethylbenzimidazole [(DMB), but purines, phenolic compounds, and other substituted benzimidazoles have also been found as cobamide lower ligands precursors found in variants of coenzyme B12 in some organisms (Hazra *et al*., 2015)], completes the coenzyme. Back in 2012, most of the anaerobic route was known but the final step, synthesis of DMB was still unknown. This was resolved in 2015 with the identification in *Eubacterium limosum* of a five‐gene operon, *bzaABCDE*, which, when expressed in *E. coli* in the absence of oxygen, synthesized not only the ligand but precursors that are used as axial ligands in a variety of forms of B12 (Moore and Warren, 2012; Hazra *et al*., 2015).

Synthesis of this ligand appeared as an interesting possibility for finding signatures, as BzaA and BzaB are specific steps involved in DMB synthesis. Both are, however, similar to the second half of the ThiC enzyme involved in the pyrimidine moiety of thiamine. Finding accurate signatures of this final step will therefore require some work to design primers for metagenome exploration. This may also be difficult for enzymes that belong to large classes of activities (such as oxidoreductase, acyltransferases or methylases). Fortunately, in the case of cobalamin, even enzymes from these ubiquitous classes must bind derivatives of the cobalt containing corrin cycle, a highly unusual substrate. Monitoring cobalamin synthesis via the sequence of cognate enzymes is thus likely to be fairly straightforward. Indeed, the cobalt‐binding corrin cycle displays highly specific features that constrain considerably the 3D structure, and likely the sequence, of proteins, enzymes and transporters that must interact with it. Using reverse translation (Mullan and Bleasby, 2002) to identify signatures to search for these signatures in metagenomes of healthy or diseased individuals (with obesity, or digestive diseases), it is possible to identify and quantify the presence of bacteria capable of synthesizing the vitamin (Table 1), then to use those to investigate the composition of known microbiomes.

**Table 1:** Protein signatures of the core anaerobic synthesis of coenzyme B12 (Fig. 3)

Enzyme	Signature
CbiA	KXGPD[YF][IL]DXX[HL]XX[AIV]
CbiC	G[FIVL][PA]VGF[IV][SG][AT]XE[SC]K
CbiD	KD[AG]GDDXD[AV]TH[GT][AML]
CbiE	ASG[ED]PLX[FY]G[AI][AG]XXL
CbiF	V[HYW][FV][IV]GAGPG[AD]X[ED]L[IL]T[LV][RK]G
CbiG	VISLLSGH[AILV]GGANX[LV]T
CbiH	D[FY][AC]X[IML]SLSDX[ML]X[SP]WXX[IV]XXR
CbiJ	[LV][AV]RVLP[TV][SA]EV[IL][IQ]XCXXLG
CbiK	P[FL]MLVAGDHA[IT]NDM[AS][SG]D[ED]X[DG]SW
CbiL	GXXV[AG]F[AIV]T[LE]GD[AP][MS][ILV][FY]ST
CbiT	D[IV]G[AS]G[TS]GSV[AST][ILV][EQ]AAXXXP
CobH	GX[PA]VGF[IV][GS][AST]X[EQ][AS]K
CobI	[HY]LXAGRX[ILV][AG][AILV][ILV]XEGD[AP][LF]F[FY][GS]S[FY][ILVM][H][MLIV]
CobJ	GAP[LIV]XXDFC[AHT][IM]SLSD
CobL	DGQ[LI]TKXX[IV]R[AV][AVIL]TL[AS]XL[AR]PXX[GS][EQ]XLWD

## Overview of relevant *Homo sapiens* microbiomes

With the prerequisite that sequence data are linked to rich structured metadata, identification of signatures of B12 synthesis in metagenomes from populations of different environments should provide worthy information. For example, the Inuit diet and lifestyle is associated with a distinct microbiome (Girard *et al*., 2017). Another relevant population is that of contemporary hunter‐gatherers (Crittenden and Schnorr, 2017) as well as populations living in populations with traditional ways of life. The Hadza people from Tanzania have provided important studies for some time (Soverini *et al*., 2016). It is, however, again, essential to be able to identify confounding factors, such as age, detailed type of food, or sampling methods, in the corresponding analyses (see Fig. 1 for a flow chart of essential steps to produce meaningful studies). Interestingly, the Hadza gut microbiome is enriched in genes involved in branched‐chain amino acid catabolic pathways via propionyl‐CoA carboxylase, ultimately producing propionate. This activity is often considered as a marker of carbon assimilation from the breakdown of complex macromolecules in the diet, providing precursors for gluconeogenesis through vitamin B12‐dependent enzyme activity (Rampelli *et al*., 2015). This does not give us conclusive clues, however, about the origin of the coenzyme.

To make more of existing microbiomes, using the signatures listed in Table 1, a preliminary study was undertaken (Braham, 2015), based on the gut microbiome of the Matses (24 persons), a remote hunter‐gatherer population from the Peruvian Amazon, the Tunapuco (12 persons), a traditional agricultural community from the Andean highlands, and residents of Norman, Oklahoma (22 persons), a typical US university community served as a comparative population following an urban‐industrialized lifestyle. Microbiomes of these groups differed significantly in terms of bacterial genome species. The authors of the corresponding analyses noticed a difference between the content in genes coding for two enzymes of B12 synthesis (a cobaltochelatase, presumably of the anaerobic pathway, and adenosylcobyric acid synthase) in the microbiome of the inhabitants of Norman (USA) and in that of the Matses and Tunapuco, the latter having more of the genes (Obregon‐Tito *et al*., 2015). However their analysis did not disclose the absolute numbers of relevant signatures, and the many further signatures of cobalamin synthesis were entirely missing from the survey. Analysis of signatures spanning the whole of anaerobic B12 synthesis showed that, as reported, the microbiome metagenomes of these populations displayed a very low number of B12 synthesis signatures (from 0 to 20, 7 per sample on average). Several of the collected samples did not even have a single signature of the coenzyme synthesis (Braham, 2015). Furthermore, the Matses consume fish as their primary meat source, animals usually rich in B12. However, it is difficult to draw firm conclusions: the study sample was quite small and it mixed up individuals (male or female more or less equally) of widely variable ages (from 1 year to 63 years, average 23 years). A limited inference from this collection of samples is that human microbiota do not contribute significantly to B12 availability, at least under the conditions used to collect and sequence samples, in populations comprising very young members.

This investigation was therefore further expanded to samples isolated in Europe (Qin *et al*., 2010), mainly from persons older than 40 years. In that second study, which grouped 124 persons (older than 18 years, average 52 years), the number of B12 signatures in the metagenomes was considerably higher (from 0 to more than 300, 78 on average). This large difference may in part be accounted for by the age structure of the samples, as well as due to differences in the feeding habits, but other causes (including host genetic set‐up, diseases and possibly sampling artefacts: replication of sampling is seldom performed in current metagenome studies, see Fig. 1) may also be at work. Again there was no strong indication of significant synthesis of cobalamin in those microbiomes, except perhaps a small anticorrelation with age of B12‐producing bacteria (this might be related to the known B12 deficiency linked to ageing). It should be noticed, however, again that several members of the cohort did not display any B12 signature (Braham, 2015).

The main outcome of these studies is that there is a considerable variation in the cobalamin synthesis capacity in different individuals, with no clear correlation with lifestyle or health status, at least within the limit of the available studies. Not surprisingly, this indicates that the vitamin, when needed, is obtained via consumption of specific animal food, from animals where the microbiota is essential to provide a significant amount of the molecule. This indicates that the role of microbiota in cobalamins synthesis may differ considerably between species. We have seen the role of coprophagy. There may exist other behaviours supplying adequate amounts of the coenzyme for specific enzyme activities. A final constraint, as we now see, must be taken into account. Cobalamin must be synthesized by specific microbiota. Yet microbes tend to limit as much as possible the production of this genetically and metabolically costly coenzyme, and they do not export it in their environment. How is this conundrum solved?

## Cobalamins as effectors of microbiota structuration

While animals have a very limited set of enzymes where coenzyme B12 is essential, this is not so for microbiota. The list of B12‐dependent enzymes in the microbial world is still growing. Besides the ubiquitous methionine synthase and methylmalonyl‐CoA mutase, B12 is essential for metabolism of propanediols (Chowdhury *et al*., 2015; Liu *et al*., 2016), ethanolamine (Jones *et al*., 2015), aminomutases (Maity *et al*., 2014) or some ribonucleoside diphosphate reductases (Taga and Walker, 2010). Recently, its essential role for epoxyqueuosine reductase activity in several bacterial clades has been deciphered (Payne *et al*., 2015). Yet, only a very limited set of prokaryotes are able to synthesize cobalamins. This places this family of molecules at the core of microbial interactions. Indeed, B12 contributes to the microbiota as a modulator of the gut microbial ecology (Degnan *et al*., 2015). Microbiota are open communities. They are also, in the gut, undergoing a more or less continuous flow, in parallel with discontinuous change in nutrient availability. On the skin, despite constant erosion microbiota also maintain functional stability (Baldwin *et al*., 2017). As a consequence, the very fact that they seem to keep significant integrity, even in the long time (Greenhalgh *et al*., 2016), shows that the microbes that compose them communicate together efficiently. This communication is not solely resulting from selection by the host immune system (Kubinak and Round, 2016) or using exchange processes such as siderophores or quorum sensing (Braga *et al*., 2016), but also via formation of a consistent meta‐metabolism (Whitfield, 2004).

This compartmentalized process supposes that metabolites get out of particular microbes to get into other ones. A major consequence of this exchange of matter is that meta‐metabolic pathways will give structure to the microbiota. Vitamin availability may drive selection for vitamin dependence, linking an organism's metabolism to its environment. In the rat, faecal communities depend on nutrients that operate considerable shifts between Firmicutes and Bacteroidetes, for example (Kalmokoff *et al*., 2015). An evolutionary experimental set‐up has demonstrated that vitamin B12 availability could play a key role in microbial genome evolution (Helliwell *et al*., 2015). Exchange of vitamin B12 has been demonstrated in the formation of a stable exchange between algae and bacteria (Grant *et al*., 2014). A major partner of human microbiota, *Bacteroides thetaiotaomicron*, is a net consumer of B12 and this allows it to communicate with other bacteria, in particular in the control of pathogens (Cordonnier *et al*., 2016). The corresponding internal competition within the gut between microbes of the *B. thetaiotaomicron* species results in a considerable alteration of the structure of the microbiota (Degnan *et al*., 2014). This is not always beneficial for the host. Microbes can scavenge the vitamin for their own usage rather than produce it for the benefit of their host. In germ‐free rats, introduction of intestinal microorganisms depletes the body B12 store of the animal, leading to signs of deficiencies (Chen and Oace, 1979). Given the importance of cobamides in environmental, industrial and human‐associated microbial metabolism, the ability to predict cobalamin synthesizing pathways in microbiomes may lead to an improved ability to understand and manipulate microbial metabolism.

Remarkably, all these observations take for granted that B12 will be freely available in the environment. Yet, because B12 synthesis is a significant genetic and metabolic burden, bacteria code for salvage systems (de Crecy‐Lagard *et al*., 2012), highly specific and sensitive importers, and rare export systems (Romine *et al*., 2017). They also display stringent control on the synthesis of the molecule as well as that of the enzymes that use it. Indeed, B12 riboswitches are widespread in prokaryotes (Nahvi *et al*., 2004). They are among the most common switches in microbial communities (Kazanov *et al*., 2007). So, how do the bacteria get a supply of B12? As for the host, a major source is animal food. Yet, this is indirect, because B12 is still from microbial origin. The consequence is that the major supplies of B12 must come from lysed microorganisms. In bacteria, the ubiquitous bacteriophage lytic/lysogenic cycle leads to occasional cell lysis, liberating its B12 content in the environment. Bacteriocins also lead to cell death and free their metabolite content. Metagenome studies demonstrate that rich microbiota are replete with phages (Debarbieux, 2014) and baceriocins (Embree *et al*., 2015). Finally, the process of sporulation is also a widespread way to spread the content of bacteria in the environment. When the spore is liberated, the mother cell, which participated in its generation, lyses. Many bacterial clades represented at a high level in gut microbiota comprise a rich collection of sporulating organisms (e.g. Clostridiales, Bacilli in particular). The lesson is that it is not enough to identify genes in microbiomes to stress that products of the corresponding metabolic pathways are directly available to the hosts. We must also provide a plausible explanation of the way B12 is liberated in the environment.

## A challenging conclusion

Cobalamin biosynthesis genes can easily be identified in microbiomes. They should be used as a reference baseline each time investigators expect (or predict) some contribution of other metabolic pathways coded in microbiomes of interest to the metabolism of their hosts (Fig. 1). Indeed, the same questions as those asked when investigating this particular, but essential metabolism, should always be asked before undertaking any study, and in particular studies with biotechnology or medical goals. Among the obvious constraints are identification of essential metadata, such as metal availability (we have seen how cobalt availability shapes B12 synthesis), bacteriophage structure of the metagenomes, or specific behaviours of the hosts, such as coprophagy. Man evolved eating lean red meat (Mann, 2000) and this allowed this species to dispense on eating faeces, helping it to put aside a heavier load of parasites. It also developed the usage of fermented food, where lactic acid bacteria provide a variety of essential vitamins (LeBlanc *et al*., 2012). Bacilli are also frequent components of fermented food, in particular in Asia (see, e.g. Bal *et al*. (2017)), and they are sporulating, which immediately liberates the cytoplasmic content of the mother cells. The essential role of lysis in the useful metabolite syntheses by microbiota is, however, challenged by recent industrial ways of mass producing fermented products, where companies try to prevent lysis as much as possible. This is illustrated by the fact that scientists from a food company discovered the antiviral immunity of the acquired CRISPR/Cas system (Horvath and Barrangou, 2010). In view of what is discussed in the present paper, one should investigate seriously whether this modern practice of preventing phage infections in fermented food may contribute to dysbiosis, rather than work as providing authentic probiotic nutrients.

## References

[mbt212722-bib-0001] Ainala, S.K. , Somasundar, A. , and Park, S. (2013) Complete genome sequence of *Pseudomonas denitrificans* ATCC 13867. Genome Announc 1: e00257–00213. 23723394 10.1128/genomeA.00257-13 PMC3668002

[mbt212722-bib-0002] Al‐Habsi, K. , Johnson, E.H. , Kadim, I.T. , Srikandakumar, A. , Annamalai, K. , Al‐Busaidy, R. , and Mahgoub, O. (2007) Effects of low concentrations of dietary cobalt on liveweight gains, haematology, serum vitamin B(12) and biochemistry of Omani goats. Vet J 173: 131–137. 16324857 10.1016/j.tvjl.2005.10.002

[mbt212722-bib-0003] Alpers, D.H. (2016) Absorption and blood/cellular transport of folate and cobalamin: pharmacokinetic and physiological considerations. Biochimie 126: 52–56. 26586110 10.1016/j.biochi.2015.11.006 PMC4867132

[mbt212722-bib-0004] Alpers, D.H. , and Russell‐Jones, G. (2013) Gastric intrinsic factor: the gastric and small intestinal stages of cobalamin absorption. A personal journey. Biochimie 95: 989–994. 23274574 10.1016/j.biochi.2012.12.006

[mbt212722-bib-0005] Bal, J. , Yun, S.H. , Yeo, S.H. , Kim, J.M. , Kim, B.T. , and Kim, D.H. (2017) Effects of initial moisture content of Korean traditional wheat‐based fermentation starter nuruk on microbial abundance and diversity. Appl Microbiol Biotechnol 101: 2093–2106. 27975136 10.1007/s00253-016-8042-2

[mbt212722-bib-0006] Baldwin, H.E. , Bhatia, N.D. , Friedman, A. , Eng, R.M. , and Seite, S. (2017) The role of cutaneous microbiota harmony in maintaining a functional skin barrier. J Drugs Dermatol 16: 12–18. 28095528

[mbt212722-bib-0007] Best, A.A. , Porter, A.L. , Fraley, S.M. , and Fraley, G.S. (2016) Characterization of gut microbiome dynamics in developing pekin ducks and impact of management system. Front Microbiol 7: 2125. 28101086 10.3389/fmicb.2016.02125 PMC5209349

[mbt212722-bib-0008] Bito, T. , and Watanabe, F. (2016) Biochemistry, function, and deficiency of vitamin B12 in *Caenorhabditis elegans* . Exp Biol Med (Maywood) 241: 1663–1668. 27486161 10.1177/1535370216662713 PMC4999627

[mbt212722-bib-0009] Bogard, J.R. , Marks, G.C. , Mamun, A. , and Thilsted, S.H. (2017) Non‐farmed fish contribute to greater micronutrient intakes than farmed fish: results from an intra‐household survey in rural Bangladesh. Public Health Nutr 20: 702–711. 27702421 10.1017/S1368980016002615 PMC5468797

[mbt212722-bib-0010] Braekkan, O.R. (1958) Vitamin B12 in marine fish. Nature 182: 1386. 10.1038/1821386a0 13600339

[mbt212722-bib-0011] Braga, R.M. , Dourado, M.N. , and Araujo, W.L. (2016) Microbial interactions: ecology in a molecular perspective. Braz J Microbiol 47(Suppl. 1): 86–98. 27825606 10.1016/j.bjm.2016.10.005 PMC5156507

[mbt212722-bib-0012] Braham, S. (2015). Exploration de signatures métaboliques spécifiques de la voie de biosynthèse de la vitamine B12 dans le génome de bactéries modèles et dans une famille de métagénomes. In Bioinformatique, Biochimie structurale et Génomique (Paris, Aix‐Marseille), pp. 62.

[mbt212722-bib-0013] Brune, A. , and Dietrich, C. (2015) The gut microbiota of termites: digesting the diversity in the light of ecology and evolution. Annu Rev Microbiol 69: 145–166. 26195303 10.1146/annurev-micro-092412-155715

[mbt212722-bib-0014] Brzuszkiewicz, E. , Waschkowitz, T. , Wiezer, A. , and Daniel, R. (2012) Complete genome sequence of the B12‐producing *Shimwellia blattae* strain DSM 4481, isolated from a cockroach. J Bacteriol 194: 4436. 22843577 10.1128/JB.00829-12 PMC3416266

[mbt212722-bib-0015] Calvani, R. , Brasili, E. , Pratico, G. , Capuani, G. , Tomassini, A. , Marini, F. , *et al* (2013) Fecal and urinary NMR‐based metabolomics unveil an aging signature in mice. Exp Gerontol 49: 5–11. 24184118 10.1016/j.exger.2013.10.010

[mbt212722-bib-0016] Caspi, R. , Billington, R. , Ferrer, L. , Foerster, H. , Fulcher, C.A. , Keseler, I.M. , *et al* (2016) The MetaCyc database of metabolic pathways and enzymes and the BioCyc collection of pathway/genome databases. Nucleic Acids Res 44: D471–D480. 26527732 10.1093/nar/gkv1164 PMC4702838

[mbt212722-bib-0017] Castle, W.B. (1985) Grain‐fed pigeons revisited: a pioneer test for vitamin B12. Br J Exp Pathol 66: 503–510. 3896291 PMC2041091

[mbt212722-bib-0018] Chen, S.C. , and Oace, S.M. (1979) Methylmalonic acid metabolism of germfree and conventional vitamin B‐12 deprived rats fed precursors of methylmalonate. J Nutr 109: 1205–1213. 571902 10.1093/jn/109.7.1205

[mbt212722-bib-0019] Chowdhury, C. , Chun, S. , Pang, A. , Sawaya, M.R. , Sinha, S. , Yeates, T.O. , and Bobik, T.A. (2015) Selective molecular transport through the protein shell of a bacterial microcompartment organelle. Proc Natl Acad Sci USA 112: 2990–2995. 25713376 10.1073/pnas.1423672112 PMC4364225

[mbt212722-bib-0020] Combes, S. , Gidenne, T. , Cauquil, L. , Bouchez, O. , and Fortun‐Lamothe, L. (2014) Coprophagous behavior of rabbit pups affects implantation of cecal microbiota and health status. J Anim Sci 92: 652–665. 24398828 10.2527/jas.2013-6394

[mbt212722-bib-0021] Coon, K.L. , Vogel, K.J. , Brown, M.R. , and Strand, M.R. (2014) Mosquitoes rely on their gut microbiota for development. Mol Ecol 23: 2727–2739. 24766707 10.1111/mec.12771 PMC4083365

[mbt212722-bib-0022] Cordonnier, C. , Le Bihan, G. , Emond‐Rheault, J.G. , Garrivier, A. , Harel, J. and Jubelin, G. (2016) Vitamin B12 uptake by the gut commensal bacteria *Bacteroides thetaiotaomicron* limits the production of Shiga toxin by enterohemorrhagic *Escherichia coli* . Toxins (Basel) 8: E14. 26742075 10.3390/toxins8010014 PMC4728536

[mbt212722-bib-0023] de Crecy‐Lagard, V. , Forouhar, F. , Brochier‐Armanet, C. , Tong, L. , and Hunt, J.F. (2012) Comparative genomic analysis of the DUF71/COG2102 family predicts roles in diphthamide biosynthesis and B12 salvage. Biol Direct 7: 32. 23013770 10.1186/1745-6150-7-32 PMC3541065

[mbt212722-bib-0024] Crisol‐Martinez, E. , Stanley, D. , Geier, M.S. , Hughes, R.J. and Moore, R.J. (2017) Understanding the mechanisms of zinc bacitracin and avilamycin on animal production: linking gut microbiota and growth performance in chickens. Appl Microbiol Biotechnol (In press). doi: 10.1007/s00253‐017‐8193‐9 10.1007/s00253-017-8193-9 28243710

[mbt212722-bib-0025] Crittenden, A.N. , and Schnorr, S.L. (2017) Current views on hunter‐gatherer nutrition and the evolution of the human diet. Am J Phys Anthropol 162(Suppl. 63): 84–109. 28105723 10.1002/ajpa.23148

[mbt212722-bib-0026] Croft, M.T. , Lawrence, A.D. , Raux‐Deery, E. , Warren, M.J. , and Smith, A.G. (2005) Algae acquire vitamin B12 through a symbiotic relationship with bacteria. Nature 438: 90–93. 16267554 10.1038/nature04056

[mbt212722-bib-0027] Danchin, A. (1973) Biological macromolecules labelling with covalent complexes of magnesium analogs. I. The cobaltic Co 3 ion. Biochimie 55: 17–27. 4720718 10.1016/s0300-9084(73)80232-9

[mbt212722-bib-0028] Danchin, A. (2010) Motivated research. EMBO Rep 11: 488. 20588310 10.1038/embor.2010.85 PMC2897119

[mbt212722-bib-0029] Debarbieux, L. (2014) Bacterial sensing of bacteriophages in communities: the search for the Rosetta stone. Curr Opin Microbiol 20: 125–130. 24952283 10.1016/j.mib.2014.05.015

[mbt212722-bib-0030] Degnan, P.H. , Barry, N.A. , Mok, K.C. , Taga, M.E. , and Goodman, A.L. (2014) Human gut microbes use multiple transporters to distinguish vitamin B(1)(2) analogs and compete in the gut. Cell Host Microbe 15: 47–57. 24439897 10.1016/j.chom.2013.12.007 PMC3923405

[mbt212722-bib-0031] Degnan, P.H. , Taga, M.E. , and Goodman, A.L. (2015) Vitamin B12 as a modulator of gut microbial ecology. Cell Metab 20: 769–778. 10.1016/j.cmet.2014.10.002 PMC4260394 25440056

[mbt212722-bib-0032] Dogan, K. , Homan, J. , Aarts, E.O. , de Boer, H. , van Laarhoven, C.J. and Berends, F.J. (2017) Long‐term nutritional status in patients following Roux‐en‐Y gastric bypass surgery. Clin Nutr (in press). doi: 10.1016/j.clnu.2017.01.022 10.1016/j.clnu.2017.01.022 28202272

[mbt212722-bib-0033] Drennan, C.L. , Matthews, R.G. , and Ludwig, M.L. (1994) Cobalamin‐dependent methionine synthase: the structure of a methylcobalamin‐binding fragment and implications for other B12‐dependent enzymes. Curr Opin Struct Biol 4: 919–929. 7712296 10.1016/0959-440x(94)90275-5

[mbt212722-bib-0034] Embree, M. , Liu, J.K. , Al‐Bassam, M.M. , and Zengler, K. (2015) Networks of energetic and metabolic interactions define dynamics in microbial communities. Proc Natl Acad Sci USA 112: 15450–15455. 26621749 10.1073/pnas.1506034112 PMC4687543

[mbt212722-bib-0035] Enck, P. , Zimmermann, K. , Rusch, K. , Schwiertz, A. , Klosterhalfen, S. , and Frick, J.S. (2009) The effects of ageing on the colonic bacterial microflora in adults. Z Gastroenterol 47: 653–658. 19606407 10.1055/s-0028-1109055

[mbt212722-bib-0036] Frese, S.A. , Benson, A.K. , Tannock, G.W. , Loach, D.M. , Kim, J. , Zhang, M. , *et al* (2011) The evolution of host specialization in the vertebrate gut symbiont *Lactobacillus reuteri* . PLoS Genet 7: e1001314. 21379339 10.1371/journal.pgen.1001314 PMC3040671

[mbt212722-bib-0037] Ghali, I. , Shinkai, T. , and Mitsumori, M. (2016) Mining of *luxS* genes from rumen microbial consortia by metagenomic and metatranscriptomic approaches. Anim Sci J 87: 666–673. 26277986 10.1111/asj.12476

[mbt212722-bib-0038] Ghosh, S. , Sinha, J.K. , Muralikrishna, B. , Putcha, U.K. and Raghunath, M. (2017) Chronic transgenerational vitamin B12 deficiency of severe and moderate magnitudes modulates adiposity‐probable underlying mechanisms. BioFactors (In press) doi: 10.1002/biof.1350 10.1002/biof.1350 28186655

[mbt212722-bib-0039] Girard, C. , Tromas, N. , Amyot, M. and Shapiro, B.J. (2017) Gut microbiome of the canadian arctic Inuit. mSphere 2: e00297–16. 28070563 10.1128/mSphere.00297-16 PMC5214747

[mbt212722-bib-0040] Grant, M.A. , Kazamia, E. , Cicuta, P. , and Smith, A.G. (2014) Direct exchange of vitamin B12 is demonstrated by modelling the growth dynamics of algal‐bacterial cocultures. ISME J 8: 1418–1427. 24522262 10.1038/ismej.2014.9 PMC4069406

[mbt212722-bib-0041] Greenhalgh, K. , Meyer, K.M. , Aagaard, K.M. , and Wilmes, P. (2016) The human gut microbiome in health: establishment and resilience of microbiota over a lifetime. Environ Microbiol 18: 2103–2116. 27059297 10.1111/1462-2920.13318 PMC7387106

[mbt212722-bib-0042] Halarnkar, P.P. , and Blomquist, G.J. (1989) Comparative aspects of propionate metabolism. Comp Biochem Physiol B 92: 227–231. 2647392 10.1016/0305-0491(89)90270-8

[mbt212722-bib-0043] Hale, V.L. , Tan, C.L. , Niu, K. , Yang, Y. , Cui, D. , Zhao, H. , *et al* (2016) Effects of field conditions on fecal microbiota. J Microbiol Methods 130: 180–188. 27686380 10.1016/j.mimet.2016.09.017

[mbt212722-bib-0044] Hanning, I. , and Diaz‐Sanchez, S. (2015) The functionality of the gastrointestinal microbiome in non‐human animals. Microbiome 3: 51. 26552373 10.1186/s40168-015-0113-6 PMC4640220

[mbt212722-bib-0045] Hazra, A.B. , Han, A.W. , Mehta, A.P. , Mok, K.C. , Osadchiy, V. , Begley, T.P. , and Taga, M.E. (2015) Anaerobic biosynthesis of the lower ligand of vitamin B12. Proc Natl Acad Sci USA 112: 10792–10797. 26246619 10.1073/pnas.1509132112 PMC4553811

[mbt212722-bib-0046] Helliwell, K.E. , Wheeler, G.L. , Leptos, K.C. , Goldstein, R.E. , and Smith, A.G. (2011) Insights into the evolution of vitamin B12 auxotrophy from sequenced algal genomes. Mol Biol Evol 28: 2921–2933. 21551270 10.1093/molbev/msr124

[mbt212722-bib-0047] Helliwell, K.E. , Collins, S. , Kazamia, E. , Purton, S. , Wheeler, G.L. , and Smith, A.G. (2015) Fundamental shift in vitamin B12 eco‐physiology of a model alga demonstrated by experimental evolution. ISME J 9: 1446–1455. 25526368 10.1038/ismej.2014.230 PMC4430296

[mbt212722-bib-0048] Horvath, P. , and Barrangou, R. (2010) CRISPR/Cas, the immune system of bacteria and archaea. Science (New York, NY) 327: 167–170. 10.1126/science.1179555 20056882

[mbt212722-bib-0049] Ibrahim, M. , and Anishetty, S. (2012) A meta‐metabolome network of carbohydrate metabolism: interactions between gut microbiota and host. Biochem Biophys Res Commun 428: 278–284. 23085046 10.1016/j.bbrc.2012.10.045

[mbt212722-bib-0050] Jacobson, S.L. , Ross, S.R. , and Bloomsmith, M.A. (2016) Characterizing abnormal behavior in a large population of zoo‐housed chimpanzees: prevalence and potential influencing factors. PeerJ 4: e2225. 27478710 10.7717/peerj.2225 PMC4950552

[mbt212722-bib-0051] Jaffe, J.J. , and Chrin, L.R. (1979) De novo synthesis of methionine in normal and Brugia‐infected Aedes aegypti. J Parasitol 65: 550–554. 512751

[mbt212722-bib-0052] Jami, E. , Israel, A. , Kotser, A. , and Mizrahi, I. (2013) Exploring the bovine rumen bacterial community from birth to adulthood. ISME J 7: 1069–1079. 23426008 10.1038/ismej.2013.2 PMC3660679

[mbt212722-bib-0053] Jones, A.R. , Rentergent, J. , Scrutton, N.S. , and Hay, S. (2015) Probing reversible chemistry in coenzyme B12 ‐dependent ethanolamine ammonia lyase with kinetic isotope effects. Chemistry 21: 8826–8831. 25950663 10.1002/chem.201500958 PMC4497352

[mbt212722-bib-0054] Kalmokoff, M. , Franklin, J. , Petronella, N. , Green, J. , and Brooks, S.P. (2015) Phylum level change in the cecal and fecal gut communities of rats fed diets containing different fermentable substrates supports a role for nitrogen as a factor contributing to community structure. Nutrients 7: 3279–3299. 25954902 10.3390/nu7053279 PMC4446752

[mbt212722-bib-0055] Kazamia, E. , Czesnick, H. , Nguyen, T.T. , Croft, M.T. , Sherwood, E. , Sasso, S. , *et al* (2012) Mutualistic interactions between vitamin B12 ‐dependent algae and heterotrophic bacteria exhibit regulation. Environ Microbiol 14: 1466–1476. 22463064 10.1111/j.1462-2920.2012.02733.x

[mbt212722-bib-0056] Kazanov, M.D. , Vitreschak, A.G. , and Gelfand, M.S. (2007) Abundance and functional diversity of riboswitches in microbial communities. BMC Genom 8: 347. 10.1186/1471-2164-8-347 PMC2211319 17908319

[mbt212722-bib-0057] Khaire, A. , Rathod, R. , Kale, A. , and Joshi, S. (2017) Vitamin B12 deficiency across three generations adversely influences long‐chain polyunsaturated fatty acid status and cardiometabolic markers in rats. Arch Med Res 47: 427–435. 10.1016/j.arcmed.2016.10.006 27986122

[mbt212722-bib-0058] Kohl, K.D. , and Carey, H.V. (2016) A place for host‐microbe symbiosis in the comparative physiologist's toolbox. J Exp Biol 219: 3496–3504. 27852759 10.1242/jeb.136325

[mbt212722-bib-0059] Kubinak, J.L. , and Round, J.L. (2016) Do antibodies select a healthy microbiota? Nat Rev Immunol 16: 767–774. 27818504 10.1038/nri.2016.114 PMC9004535

[mbt212722-bib-0060] Langille, M.G. , Meehan, C.J. , Koenig, J.E. , Dhanani, A.S. , Rose, R.A. , Howlett, S.E. , and Beiko, R.G. (2014) Microbial shifts in the aging mouse gut. Microbiome 2: 50. 25520805 10.1186/s40168-014-0050-9 PMC4269096

[mbt212722-bib-0061] LeBlanc, J.G. , Milani, C. , de Giori, G.S. , Sesma, F. , van Sinderen, D. , and Ventura, M. (2012) Bacteria as vitamin suppliers to their host: a gut microbiota perspective. Curr Opin Biotechnol 24: 160–168. 22940212 10.1016/j.copbio.2012.08.005

[mbt212722-bib-0062] Lee, S.G. , Kaya, A. , Avanesov, A.S. , Podolskiy, D.I. , Song, E.J. , Go, D.M. , *et al* (2017) Age‐associated molecular changes are deleterious and may modulate life span through diet. Sci Adv 3: e1601833. 28232953 10.1126/sciadv.1601833 PMC5315447

[mbt212722-bib-0063] Leulier, F. , MacNeil, L.T. , Lee, W.J. , Rawls, J.F. , Cani, P.D. , Schwarzer, M. , *et al* (2017) Integrative physiology: at the crossroads of nutrition, microbiota, animal physiology, and human health. Cell Metab 25: 522–534. 28273475 10.1016/j.cmet.2017.02.001 PMC6200423

[mbt212722-bib-0064] Li, X. , Zhou, L. , Yu, Y. , Ni, J. , Xu, W. and Yan, Q. (2017) Composition of gut microbiota in the gibel carp (*Carassius auratus gibelio*) varies with host development. Microb Ecol (In press). doi: 10.1007/s00248‐016‐0924‐4 10.1007/s00248-016-0924-4 28108758

[mbt212722-bib-0065] Liu, J.Z. , Xu, W. , Chistoserdov, A. , and Bajpai, R.K. (2016) Glycerol dehydratases: biochemical structures, catalytic mechanisms, and industrial applications in 1,3‐propanediol production by naturally occurring and genetically engineered bacterial strains. Appl Biochem Biotechnol 179: 1073–1100. 27033090 10.1007/s12010-016-2051-6

[mbt212722-bib-0066] Magnusdottir, S. , Ravcheev, D. , de Crecy‐Lagard, V. , and Thiele, I. (2015) Systematic genome assessment of B‐vitamin biosynthesis suggests co‐operation among gut microbes. Front Genet 6: 148. 25941533 10.3389/fgene.2015.00148 PMC4403557

[mbt212722-bib-0067] Maity, A.N. , Chen, Y.H. , and Ke, S.C. (2014) Large‐scale domain motions and pyridoxal‐5′‐phosphate assisted radical catalysis in coenzyme B12‐dependent aminomutases. Int J Mol Sci 15: 3064–3087. 24562332 10.3390/ijms15023064 PMC3958899

[mbt212722-bib-0068] Mann, N. (2000) Dietary lean red meat and human evolution. Eur J Nutr 39: 71–79. 10918988 10.1007/s003940050005

[mbt212722-bib-0069] Matthews, R.G. , Smith, A.E. , Zhou, Z.S. , Taurog, R.E. , Bandarian, V. , Evans, J.C. , and Ludwig, M. (2003) Cobalamin‐dependent and cobalamin‐independent methionine synthases: are there two solutions to the same chemical problem? Helv Chim Acta 86: 3939–3954.

[mbt212722-bib-0070] McCutcheon, J.P. , McDonald, B.R. , and Moran, N.A. (2009) Convergent evolution of metabolic roles in bacterial co‐symbionts of insects. Proc Natl Acad Sci USA 106: 15394–15399. 19706397 10.1073/pnas.0906424106 PMC2741262

[mbt212722-bib-0071] Milton, K. (1987) Primate diets and gut morphology: implications for hominid evolution In Food and Evolution Toward a Theory of Human Food Habits. Harris M., and Ross E.B. (eds). Philadelphia, PA, USA: Temple University Press, pp. 93–115.

[mbt212722-bib-0072] Moore, S.J. , and Warren, M.J. (2012) The anaerobic biosynthesis of vitamin B12. Biochem Soc Trans 40: 581–586. 22616870 10.1042/BST20120066

[mbt212722-bib-0073] Mullan, L.J. , and Bleasby, A.J. (2002) Short EMBOSS user guide. European Molecular Biology open software suite. Brief Bioinform 3: 92–94. 12002228 10.1093/bib/3.1.92

[mbt212722-bib-0074] Mweresa, C.K. , Mukabana, W.R. , Omusula, P. , Otieno, B. , Van Loon, J.J. , and Takken, W. (2016) Enhancing attraction of African malaria vectors to a synthetic odor blend. J Chem Ecol 42: 508–516. 27349651 10.1007/s10886-016-0711-1

[mbt212722-bib-0075] Nahvi, A. , Barrick, J.E. , and Breaker, R.R. (2004) Coenzyme B12 riboswitches are widespread genetic control elements in prokaryotes. Nucleic Acids Res 32: 143–150. 14704351 10.1093/nar/gkh167 PMC373277

[mbt212722-bib-0076] Negro, J.J. , Grande, J.M. , Tella, J.L. , Garrido, J. , Hornero, D. , Donazar, J.A. , *et al* (2002) Coprophagy: an unusual source of essential carotenoids. Nature 416: 807–808. 11976670 10.1038/416807a

[mbt212722-bib-0077] Nie, Y. , Zhou, Z. , Guan, J. , Xia, B. , Luo, X. , Yang, Y. , *et al* (2017) Dynamic changes of yak (*Bos grunniens*) gut microbiota during growth revealed by PCR‐DGGE and metagenomics. Asian‐Australas J Anim Sci (In press). doi: 10.5713/ajas.16.0836 10.5713/ajas.16.0836 PMC5495674 28183172

[mbt212722-bib-0078] Obregon‐Tito, A.J. , Tito, R.Y. , Metcalf, J. , Sankaranarayanan, K. , Clemente, J.C. , Ursell, L.K. , *et al* (2015) Subsistence strategies in traditional societies distinguish gut microbiomes. Nat Commun 6, 6505. 25807110 10.1038/ncomms7505 PMC4386023

[mbt212722-bib-0079] Odamaki, T. , Kato, K. , Sugahara, H. , Hashikura, N. , Takahashi, S. , Xiao, J.Z. , *et al* (2016) Age‐related changes in gut microbiota composition from newborn to centenarian: a cross‐sectional study. BMC Microbiol 16: 90. 27220822 10.1186/s12866-016-0708-5 PMC4879732

[mbt212722-bib-0080] Ohta, A. , Baba, S. , Ohtsuki, M. , Taguchi, A. , and Adachi, T. (1996) Prevention of coprophagy modifies magnesium absorption in rats fed with fructo‐oligosaccharides. Br J Nutr 75: 775–784. 8695604 10.1079/bjn19960181

[mbt212722-bib-0081] Ortigues‐Marty, I. , Micol, D. , Prache, S. , Dozias, D. , and Girard, C.L. (2005) Nutritional value of meat: the influence of nutrition and physical activity on vitamin B12 concentrations in ruminant tissues. Reprod Nutr Dev 45: 453–467. 16045893 10.1051/rnd:2005038

[mbt212722-bib-0082] Payne, K.A. , Fisher, K. , Sjuts, H. , Dunstan, M.S. , Bellina, B. , Johannissen, L. , *et al* (2015) Epoxyqueuosine reductase structure suggests a mechanism for cobalamin‐dependent tRNA modification. J Biol Chem 290: 27572–27581. 26378237 10.1074/jbc.M115.685693 PMC4646009

[mbt212722-bib-0083] Pejchal, R. , and Ludwig, M.L. (2005) Cobalamin‐independent methionine synthase (MetE): a face‐to‐face double barrel that evolved by gene duplication. PLoS Biol 3: e31. 15630480 10.1371/journal.pbio.0030031 PMC539065

[mbt212722-bib-0084] Perkins, G.A. , den Bakker, H.C. , Burton, A.J. , Erb, H.N. , McDonough, S.P. , McDonough, P.L. , *et al* (2012) Equine stomachs harbor an abundant and diverse mucosal microbiota. Appl Environ Microbiol 78: 2522–2532. 22307294 10.1128/AEM.06252-11 PMC3318809

[mbt212722-bib-0085] Qin, J. , Li, R. , Raes, J. , Arumugam, M. , Burgdorf, K.S. , Manichanh, C. , *et al* (2010) A human gut microbial gene catalogue established by metagenomic sequencing. Nature 464: 59–65. 20203603 10.1038/nature08821 PMC3779803

[mbt212722-bib-0086] Rampelli, S. , Schnorr, S.L. , Consolandi, C. , Turroni, S. , Severgnini, M. , Peano, C. , *et al* (2015) Metagenome sequencing of the Hadza hunter‐gatherer gut microbiota. Curr Biol 25: 1682–1693. 25981789 10.1016/j.cub.2015.04.055

[mbt212722-bib-0087] Ravanel, S. , Block, M.A. , Rippert, P. , Jabrin, S. , Curien, G. , Rebeille, F. , and Douce, R. (2004) Methionine metabolism in plants: chloroplasts are autonomous for de novo methionine synthesis and can import S‐adenosylmethionine from the cytosol. J Biol Chem 279: 22548–22557. 15024005 10.1074/jbc.M313250200

[mbt212722-bib-0088] Rizzo, G. , Lagana, A.S. , Rapisarda, A.M. , La Ferrera, G.M. , Buscema, M. , Rossetti, P. , *et al* (2016) Vitamin B12 among vegetarians: status, assessment and supplementation. Nutrients 8: E767. 27916823 10.3390/nu8120767 PMC5188422

[mbt212722-bib-0089] Robel, E.J. (1983) The effect of age of breeder hen on the levels of vitamins and minerals in turkey eggs. Poult Sci 62: 1751–1756. 6634604 10.3382/ps.0621751

[mbt212722-bib-0090] Roberts, M.C. (1983) Serum and red cell folate and serum vitamin B12 levels in horses. Aust Vet J 60: 106–111. 6870712 10.1111/j.1751-0813.1983.tb05906.x

[mbt212722-bib-0091] Romine, M.F. , Rodionov, D.A. , Maezato, Y. , Osterman, A.L. and Nelson, W.C. (2017) Underlying mechanisms for syntrophic metabolism of essential enzyme cofactors in microbial communities. ISME J (In press). doi: 10.1038/ismej.2017.2 10.1038/ismej.2017.2 PMC5437353 28186498

[mbt212722-bib-0092] Rosenberg, E. , and Zilber‐Rosenberg, I. (2016) Microbes drive evolution of animals and plants: the hologenome concept. MBio 7: e01395. 10.1128/mBio.01395-15 PMC4817260 27034283

[mbt212722-bib-0093] Sakamaki, T. (2010) Coprophagy in wild bonobos (*Pan paniscus*) at Wamba in the Democratic Republic of the Congo: a possibly adaptive strategy? Primates 51: 87–90. 19882210 10.1007/s10329-009-0167-9

[mbt212722-bib-0094] Shipton, M.J. , and Thachil, J. (2015) Vitamin B12 deficiency – A 21st century perspective. Clin Med (Lond) 15: 145–150. 25824066 10.7861/clinmedicine.15-2-145 PMC4953733

[mbt212722-bib-0095] Simard, F. , Guay, F. , Girard, C.L. , Giguere, A. , Laforest, J.P. , and Matte, J.J. (2007) Effects of concentrations of cyanocobalamin in the gestation diet on some criteria of vitamin B12 metabolism in first‐parity sows. J Anim Sci 85: 3294–3302. 17709774 10.2527/jas.2006-523

[mbt212722-bib-0096] Soave, O. , and Brand, C.D. (1991) Coprophagy in animals: a review. Cornell Vet 81: 357–364. 1954740

[mbt212722-bib-0097] Soverini, M. , Rampelli, S. , Turroni, S. , Schnorr, S.L. , Quercia, S. , Castagnetti, A. , *et al* (2016) Variations in the post‐weaning human gut metagenome profile as result of *Bifidobacterium* acquisition in the western microbiome. Front Microbiol 7: 1058. 27462302 10.3389/fmicb.2016.01058 PMC4940381

[mbt212722-bib-0098] Spinler, J.K. , Sontakke, A. , Hollister, E.B. , Venable, S.F. , Oh, P.L. , Balderas, M.A. , *et al* (2014) From prediction to function using evolutionary genomics: human‐specific ecotypes of *Lactobacillus reuteri* have diverse probiotic functions. Genome Biol Evol 6: 1772–1789. 24951561 10.1093/gbe/evu137 PMC4122935

[mbt212722-bib-0099] Tachon, S. , Zhou, J. , Keenan, M. , Martin, R. , and Marco, M.L. (2012) The intestinal microbiota in aged mice is modulated by dietary resistant starch and correlated with improvements in host responses. FEMS Microbiol Ecol 83: 299–309. 22909308 10.1111/j.1574-6941.2012.01475.x

[mbt212722-bib-0100] Taga, M.E. , and Walker, G.C. (2010) *Sinorhizobium meliloti* requires a cobalamin‐dependent ribonucleotide reductase for symbiosis with its plant host. Mol Plant Microbe Interact 23: 1643–1654. 20698752 10.1094/MPMI-07-10-0151 PMC2979309

[mbt212722-bib-0101] Tosteson, T.R. (1995) The diversity and origins of toxins in ciguatera fish poisoning. P R Health Sci J 14: 117–129. 7617831

[mbt212722-bib-0102] Toyoshima, M. , Inada, M. , and Kameyama, M. (1983) Effects of aging on intracellular transport of vitamin B12 (B12) in rat enterocytes. J Nutr Sci Vitaminol (Tokyo) 29: 1–10. 6864344 10.3177/jnsv.29.1

[mbt212722-bib-0103] Walsh, P.T. , McCreless, E. , and Pedersen, A.B. (2013) Faecal avoidance and selective foraging: do wild mice have the luxury to avoid faeces? Anim Behav 86: 559–566. 24027342 10.1016/j.anbehav.2013.06.011 PMC3763379

[mbt212722-bib-0104] Watanabe, F. , Yabuta, Y. , Tanioka, Y. , and Bito, T. (2013) Biologically active vitamin B12 compounds in foods for preventing deficiency among vegetarians and elderly subjects. J Agric Food Chem 61: 6769–6775. 23782218 10.1021/jf401545z

[mbt212722-bib-0105] Whitfield, J. (2004) Ecology's big, hot idea. PLoS Biol 2: e440. 15597118 10.1371/journal.pbio.0020440 PMC535575

[mbt212722-bib-0106] Wolthers, K.R. , and Scrutton, N.S. (2009) Cobalamin uptake and reactivation occurs through specific protein interactions in the methionine synthase‐methionine synthase reductase complex. FEBS J 276: 1942–1951. 19243433 10.1111/j.1742-4658.2009.06919.x

[mbt212722-bib-0107] Zhang, Y. , Rodionov, D.A. , Gelfand, M.S. , and Gladyshev, V.N. (2009) Comparative genomic analyses of nickel, cobalt and vitamin B12 utilization. BMC Genom 10: 78. 10.1186/1471-2164-10-78 PMC2667541 19208259

